# Supplementary material for: Exploring Echinops polyceras Boiss. from Jordan: Essential Oil Composition, COX, Protein Denaturation Inhibitory Power and Antimicrobial Activity of the Alcoholic Extract
Source: Molecules. 2023 May 22;28(10):4238. doi: 10.3390/molecules28104238 (PMC10223352; doi:10.3390/molecules28104238)
Supplement: Supplementary file 1 [file molecules-28-04238-s001.zip › molecules-2400000-supplementary.pdf]

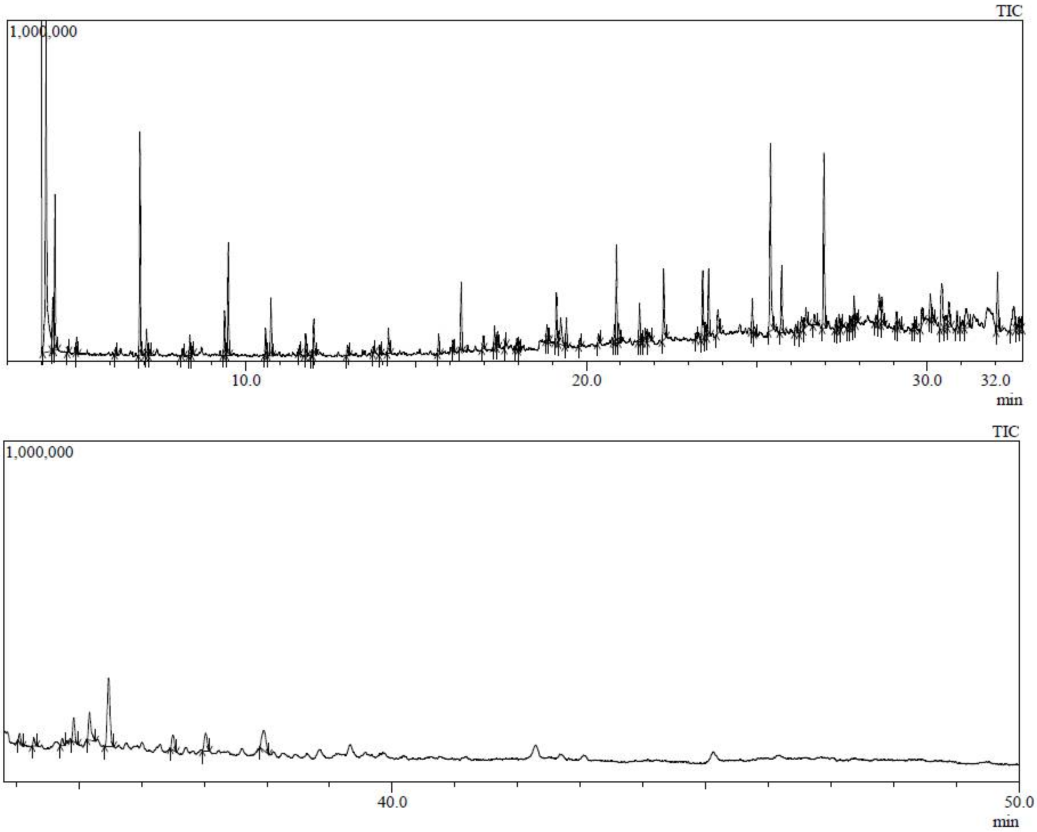

Figure S1: GC-MS analysis of essential oil during pre-flowring stage of *Echinops polyceras* Boiss. Inflorescence

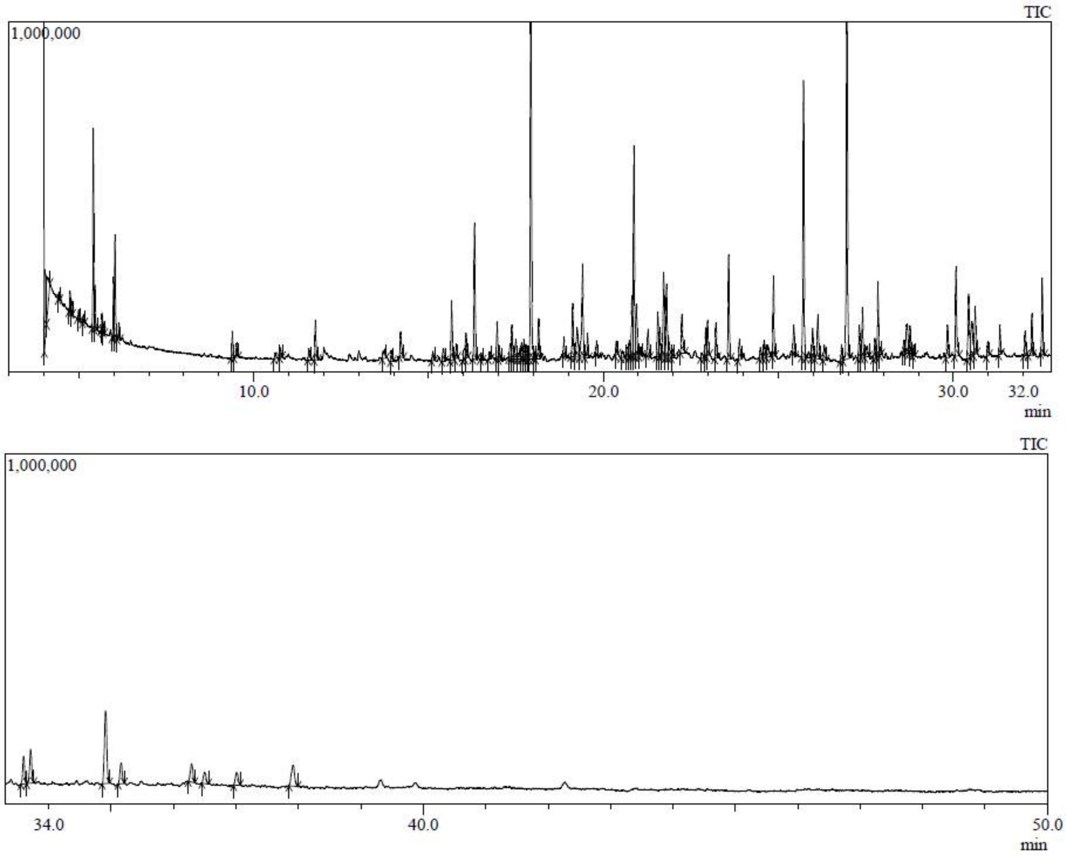

Figure S2: GC-MS analysis of essential oil during full-flowring stage of *Echinops polyceras* Boiss. Inflorescence

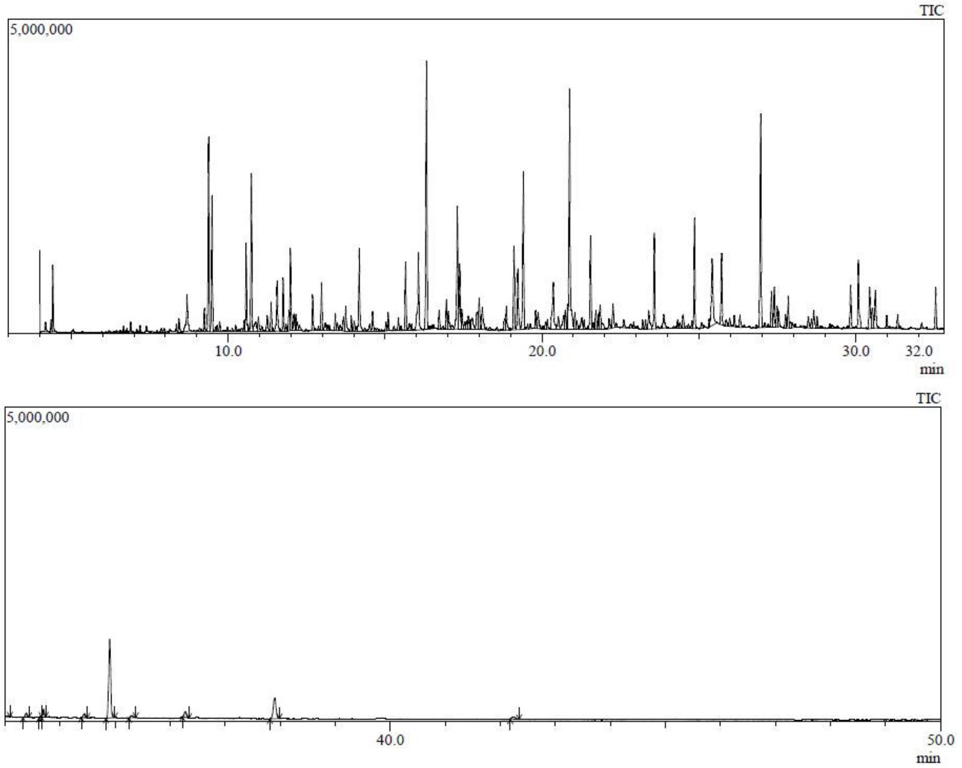

Figure S3: GC-MS analysis of essential oil during post-flowring stage of *Echinops polyceras* Boiss. inflorescence
